# Blue–Red LED Light Modulates Morphophysiological and Metabolic Responses in the Medicinal Plant *Nepeta nuda*

**DOI:** 10.3390/plants14152285

**Published:** 2025-07-24

**Authors:** Miroslava Zhiponova, Grigor Zehirov, Krasimir Rusanov, Mila Rusanova, Miroslava Stefanova, Tsveta Ganeva, Momchil Paunov, Valentina Ganeva, Kiril Mishev, Petre I. Dobrev, Roberta Vaculíková, Václav Motyka, Zhenya Yordanova, Ganka Chaneva, Valya Vassileva

**Affiliations:** 1 Faculty of Biology, Sofia University “St. Kliment Ohridski”, 1164 Sofia, Bulgaria; m.stephanova@biofac.uni-sofia.bg (M.S.); tsvetaganeva@biofac.uni-sofia.bg (T.G.); m_paunov@uni-sofia.bg (M.P.); valia@biofac.uni-sofia.bg (V.G.); jiordanova@biofac.uni-sofia.bg (Z.Y.); chaneva@biofac.uni-sofia.bg (G.C.); 2 Institute of Plant Physiology and Genetics, Bulgarian Academy of Sciences, 1113 Sofia, Bulgaria; grig@bio21.bas.bg (G.Z.); mishev@bio21.bas.bg (K.M.); 3 Agrobioinstitute, Agricultural Academy, 1164 Sofia, Bulgaria; krusanov@abv.bg (K.R.); milagradeva@abv.bg (M.R.); 4 Centre of Competence “Sustainable Utilization of Bio-Resources and Waste of Medicinal and Aromatic Plants for Innovative Bioactive Products” (BIORESOURCES BG), 1000 Sofia, Bulgaria; 5 Institute of Experimental Botany of the Czech Academy of Sciences, 165 00 Prague, Czech Republic; dobrev@ueb.cas.cz (P.I.D.); filepova@ueb.cas.cz (R.V.); vmotyka@ueb.cas.cz (V.M.)

**Keywords:** leaf anatomy, phenolic compounds, phytohormones, plastid pigments, ROS, volatiles

## Abstract

Light quality and duration profoundly influence the growth and productivity of plant species. This study investigated the effects of a blue–red LED light combination, known to induce flowering, on the physiological state and content of biologically active substances in catmint (*Nepeta nuda* L.) grown under controlled in vitro conditions. White light (W) was used as a control and compared with two blue–red intensities: BR (high-intensity blue–red light) and BRS (low-intensity blue–red light or “BR with shadow”). BR-treated plants showed increased leaf area, mesophyll thickness, biomass and starch content but reduced levels of plastid pigments. BR also modified the oxidative state of plants by inducing lipid peroxidation while simultaneously activating ROS scavenging mechanisms and enhancing phenolic antioxidants. Interestingly, BR decreased the accumulation of the *Nepeta* sp.-specific iridoid, nepetalactone. These effects appear to be regulated by the phytohormones auxin, abscisic acid and jasmonates. BRS treatment produced effects similar to the W control but led to increased plant height and reduced leaf area and thickness. Both BR and BRS regimes induced the accumulation of proteins and amino acids. We conclude that blue–red light can enhance the survival capacity of micropropagated *N. nuda* during subsequent soil adaptation, suggesting that similar light pre-treatment could improve plant performance under stress conditions.

## 1. Introduction

Light is one of the most critical environmental factors regulating plant growth, morphogenesis, physiology and metabolism [1,2,3,4,5,6]. The quality (spectrum) and quantity (intensity) of light significantly influence morphological, anatomical and photosynthetic traits [2,3,4]. Various light sources, such as light-emitting diodes (LEDs), fluorescent lamps and high-pressure sodium and metal halide lamps, can provide specific spectral compositions ranging from narrow peaks of a specific range of wavelength to broad-spectrum illumination. Among these, LEDs are considered the most energy-efficient and adaptable for controlled environment agriculture [3,4].

The biosynthesis of bioactive compounds in medicinal, aromatic and spice plants is highly responsive to light conditions [2,4]. Depending on the species and targeted metabolites, light can exert positive, neutral or even inhibitory effects [7]. Photosynthetically active radiation (PAR; 400–700 nm) and ultraviolet (UV-A: 315–400 nm; UV-B: 280–320 nm) light are recognized as key regulators of both biomass production and the synthesis of essential oils and phenolics in species such as catmint (*Nepeta cataria* L. ‘*citriodora*’), lemon balm (*Melissa officinalis* L.) and sage (*Salvia officinalis* L.) [7]. Red and blue light treatments, applied either individually or in combination, have been extensively investigated in medicinal plants, including sweet wormwood (*Artemisia annua* L.), sweet basil (*Ocimum basilicum* L.) and sage (*Salvia przewalskii* Maxim.) [2,4,8]. Furthermore, environmental factors such as irradiance, temperature, water availability and seasonal variation have demonstrated significant effects on the metabolic profiles of species such as rosemary (*Salvia rosmarinus* Spenn.) and sage (*Salvia yangii* B. T. Drew) [9,10,11].

Members of the genus *Nepeta* (Lamiaceae) are cultivated as ornamental and medicinal plants, primarily for their production of terpenes and phenolic compounds with pharmaceutical potential [12,13,14,15,16]. Many *Nepeta* species synthesize unique iridoid monoterpenes, particularly nepetalactones and their glycosides, which are known for their ecological roles in plant defense. In wild-grown *Nepeta nuda* L., high levels of secondary metabolites with antifeedant and repellent properties contribute to enhanced plant immunity [15,16,17]. Native to open meadows and forest clearings, *N. nuda* is classified as a heliophytic or ‘sun-loving’ species, indicating a preference for high light environments [15].

Comparative studies between wild-grown and in vitro-cultivated *N. nuda* have shown substantial reductions in growth and metabolic activity under controlled in vitro conditions [15,16]. As light spectrum and intensity are known to influence flowering and secondary metabolism [2,3,4], our previous work demonstrated that both flowers of wild-grown plants and a light formula for boosting flowering in in vitro conditions exhibited elevated levels of phenolic compounds [15]. In particular, high-intensity blue–red (BR) LED light significantly enhanced the accumulation of rosmarinic acid, cirsimaritin, naringenin, rutin, isoquercetin and the iridoid glycoside 1,5,9-epideoxyloganic acid. These promising findings prompted a deeper investigation into the mechanisms underlying the response of *N. nuda* to light.

The present study aimed to examine how different light spectra and intensities affect physiological responses in *N. nuda* during controlled in vitro cultivation. Specifically, we investigated how blue–red LED light treatments affect plant morphology, oxidative status, metabolic production, hormonal regulation and subsequent adaptation capacity in ex vitro conditions. Through the development of optimized light protocols for the micropropagation of medicinal plants, this research could improve survival rates and the production of bioactive compounds during the critical transition from in vitro to greenhouse conditions. Our findings are expected to improve commercial cultivation of *N. nuda* and other medicinal *Nepeta* species and contribute to a better understanding of light-mediated stress priming mechanisms applicable to economically important plants in controlled environment agriculture.

## 2. Results

### 2.1. Influence of Varied Light Regimes on Growth Parameters of In Vitro Cultivated Plants

**Figure 1:**
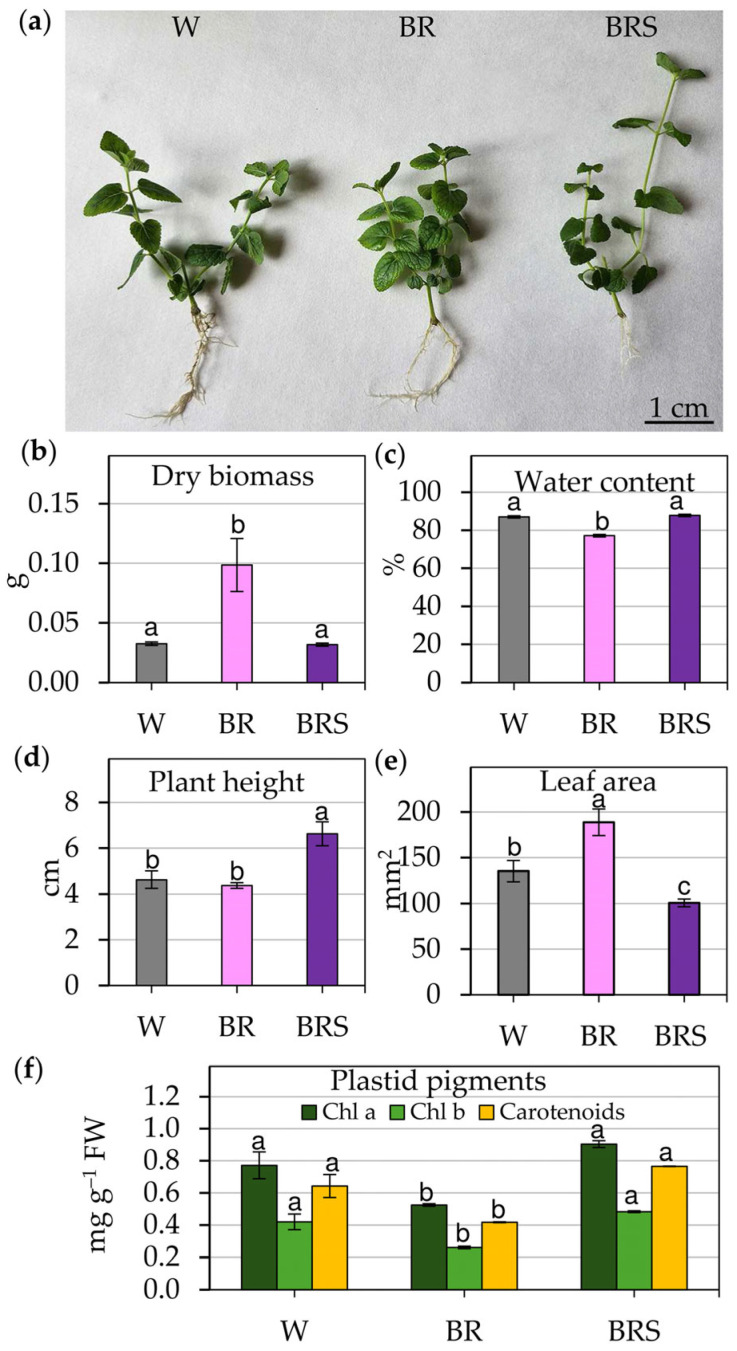
Effect of different light regimes on the growth of *N. nuda* plants in in vitro conditions. (**a**) Morphology of plants derived from mono-nodal explants cultivated under white (W), high-intensity blue–red (BR) or low-intensity blue–red (BRS) light for 5 weeks. Scale bar: 1 cm. (**b**) Dry biomass; (**c**) water content; (**d**) plant height; (**e**) leaf area. (**f**) Plastid pigment content—chlorophyll a (Chl a), chlorophyll b (Chl b) and carotenoids. Data represents mean ± SE (standard error) (*n* > 25). Statistical differences among treatments for each pigment were determined by one-way ANOVA followed by the Holm–Sidak post-hoc test; different letters indicate significant differences (*p* < 0.05).

In vitro cultivation of *N. nuda* under different light conditions resulted in distinct morphological and physiological changes (Figure 1) with noticeable phenotypic differences visible in whole plants (Figure 1a).

A combination of high-intensity blue–red light (BR) significantly increased dry biomass but decreased water content compared to white light (W) (Figure 1b,c). In contrast, low-intensity blue–red light (BRS) resulted in a pronounced increase in plant height (Figure 1d). Although BR stimulated leaf expansion and significantly increased total leaf area (Figure 1e), plants grown under BRS conditions showed reduced leaf area (Figure 1e). Water content in BRS-grown plants remained comparable to that of the W control group (Figure 1c), suggesting differential regulation of water retention mechanisms under low-intensity light. Furthermore, plastid pigment analysis revealed a substantial decline in the levels of chlorophyll a, chlorophyll b and carotenoids under high-intensity BR light, with a partial recovery of pigments observed under BRS conditions (Figure 1f).

### 2.2. Evaluation of Physiological Changes in In Vitro Cultivated Plants Under Different Light Regimes

**Figure 2:**
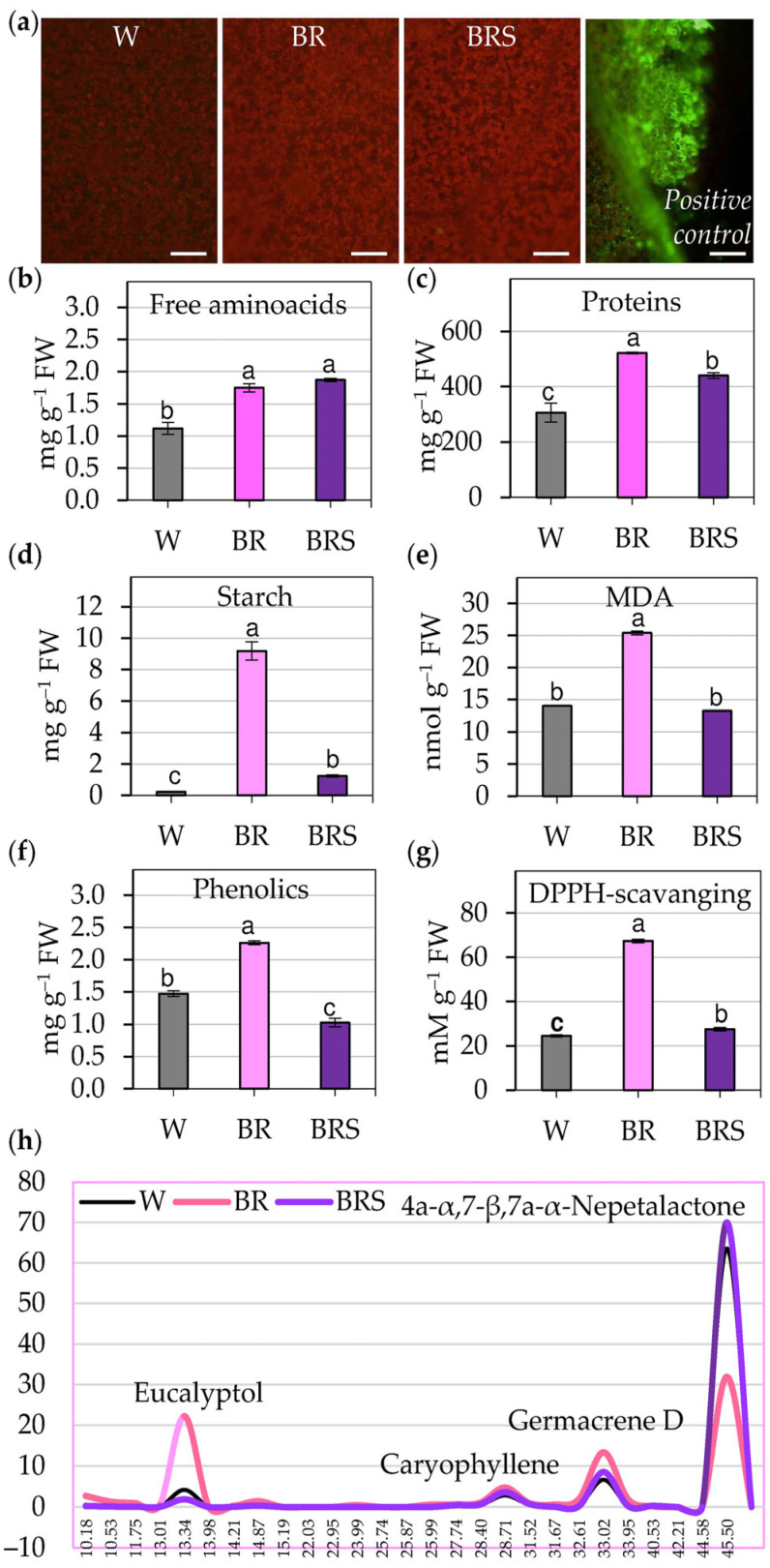
Effect of light quality and intensity on the functional status of in vitro-cultivated *N. nuda* plantlets. (**a**) ROS production visualized by DCF-DA (2′,7′-dichlorodihydrofluorescein diacetate) staining in leaf epidermis and mesophyll. Green fluorescence indicates DCF-DA signal (ROS), and red fluorescence corresponds to chlorophyll autofluorescence in mesophyll cells. A wounded leaf served as positive control. Scale bar: 10 µm. (**b**) Content of free amino acids. (**c**) Total soluble proteins. (**d**) Starch accumulation. (**e**) Lipid peroxidation level, defined by malondialdehyde (MDA) content. (**f**) Total phenolic compounds. (**g**) Antioxidant capacity assessed by DPPH (2,2-diphenyl-1-picrylhydrazyl) radical scavenging antioxidant activity. (**h**) Volatile organic compounds (including eucalyptol, caryophyllene, germacrene D, and 4a-α,7-β,7a-α-nepetalactone) identified by GC-MS (gas chromatography–mass spectrometry) analysis. Data represent mean ± SE (n > 25). Statistical differences among treatments were determined using one-way ANOVA, followed by the Holm–Sidak test; different letters indicate significant differences (*p* < 0.05). Abbreviations: W, white light; BR, blue–red light with high intensity; BRS, blue–red light with low intensity.

**Figure 3:**
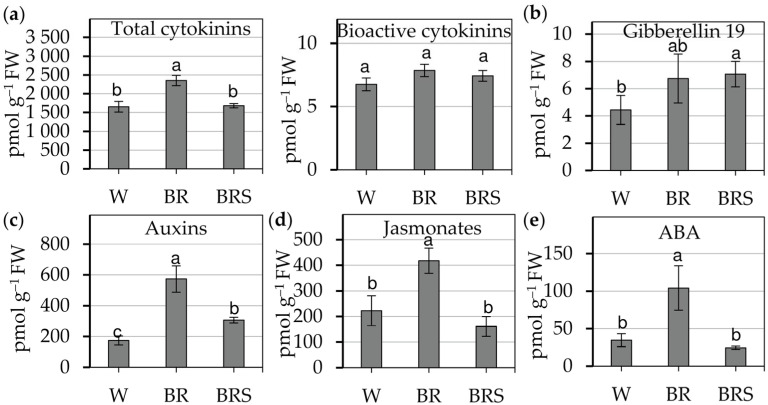
Quantification of endogenous phytohormones in *N. nuda* in vitro-cultivated plants under different light regimes. (**a**) Cytokinins (total and bioactive). (**b**) Gibberellin 19. (**c**) Auxins. (**d**) Jasmonates. (**e**) Abscisic acid (ABA). Bars represent mean values ± SE for each treatment. Statistically significant differences between treatments were assessed using one-way ANOVA followed by Holm–Sidak post-hoc test; different letters indicate significant differences (*p* < 0.05). Abbreviations: W, white light; BR, blue–red light with high intensity; BRS, blue–red light with low intensity.

To further evaluate the physiological responses of *N. nuda* to the tested different light regimes, we assessed oxidative status, productivity potential and phytohormonal regulation (Figure 2 and Figure 3).

#### 2.2.1. Oxidative State and Metabolic Content

Detection of reactive oxygen species (ROS) using DCF-DA fluorescence revealed no visible ROS accumulation in the leaf epidermis under any of the light treatments (Figure 2a and Figure S2a,b). Both BR and BRS light conditions significantly increased free amino acid (Figure 2b) and protein content (Figure 2c and Figure S1), as well as starch accumulation (Figure 2d). High-intensity BR light increased levels of lipid peroxidation (Figure 2e), indicating oxidative stress. This response was accompanied by elevated phenolic compounds and enhanced antioxidant capacity, as evidenced by DPPH scavenging activity (Figure 2f,g). Additionally, volatile secondary metabolites, such as eucalyptol, germacrene D, caryophyllene and other volatile iridoids, were upregulated under BR light with a notable decrease in the key compound 4a-α,7-β,7a-α-nepetalactone (Figure 2h; Table S1). A supplementary short-term BR treatment confirmed that ROS were generated early during exposure but were likely neutralized over time (Figure S2c,d), supporting an oxidative burst followed by compensatory antioxidant synthesis.

#### 2.2.2. Light-Induced Changes in Endogenous Phytohormones

The effect of light quality and intensity on endogenous phytohormone levels in *N. nuda* was assessed using LC-MS/MS (Figure 3).

The blue–red light regimes did not substantially affect cytokinin metabolism, as the BR treatment increased total cytokinin levels compared to white light and BRS (Figure 3a left). However, the levels of bioactive cytokinin bases, which are directly involved in signal transduction, remained unaffected across treatments (Figure 3a right). The BRS treatment led to a significant rise in gibberellin 19 levels (Figure 3b). Auxin accumulation was notably enhanced by BR light, showing its role in the growth promotion under BR (Figure 3c). Similarly, BR illumination triggered a marked increase in jasmonates and ABA (abscisic acid) levels, indicating its role in stress-related hormonal pathways (Figure 3d,e). Collectively, the data underscore the interplay between light spectrum and intensity in the regulation of phytohormonal balance, influencing growth, development and environmental responses of *N. nuda*.

### 2.3. Potential for Ex Vitro Adaptation

#### 2.3.1. Leaf Histological Parameters

**Figure 4:**
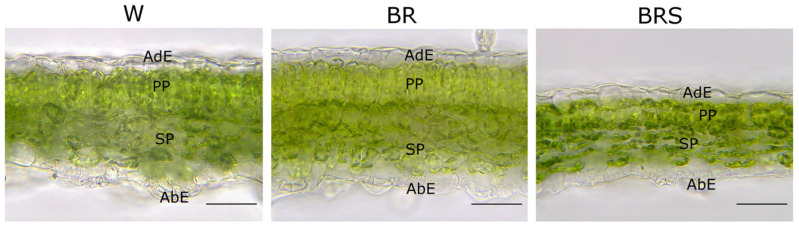
Leaf anatomy of in vitro-cultivated *N. nuda* plantlets under different light regimes. Abbreviations: W, white light; BR, blue–red light with high intensity; BRS, blue–red light with low intensity; PP, palisade parenchyma; SP, spongy parenchyma; AdE, adaxial epidermis; AbE, abaxial epidermis. Scale bar: 50 µm.

Microscopic analysis of transverse leaf sections revealed a bifacial mesophyll organization across all light treatments, consisting of a clearly distinguishable palisade parenchyma on the adaxial side and a spongy parenchyma on the abaxial side (Figure 4).

Interestingly, differences in mesophyll structure were observed under the different light regimes. Under W light, the palisade parenchyma formed a single layer of elongated cylindrical cells. A similar single-layer arrangement was seen under BRS light, but with shorter, more oval-shaped cells. In contrast, BR light induced the development of a two-layered palisade parenchyma: an upper layer of cylindrical cells and a lower layer of oval cells. The spongy parenchyma in all three treatments was consistently three-layered, made up of relatively densely packed oval cells. The epidermis on both sides of the leaf was single-layered and composed of compact cells with relatively uniform height. These qualitative observations were supported by quantitative morphometric analysis (Table 1).

Leaves developed on plantlets grown under BR light showed the greatest overall tissue thickness, with the lamina, mesophyll, palisade and spongy parenchyma layers all being approximately 25% thicker than those under W light. Notably, BR irradiation did not significantly affect the height of the epidermal cells compared to W or BRS treatments. In contrast, BRS light resulted in the thinnest leaf anatomy across all measured parameters. Compared to W light, leaves under BRS showed a 16% reduction in lamina thickness, a 21% reduction in mesophyll thickness, a 24% thinner palisade layer and a 16% thinner spongy parenchyma. No statistically significant differences were found in the thickness of the adaxial or abaxial epidermis among the treatments.

#### 2.3.2. Leaf Epidermis

**Figure 5:**
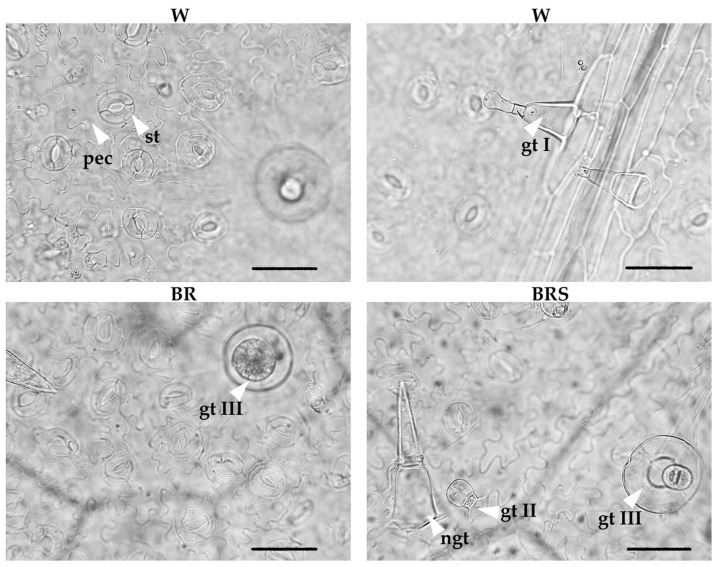
Abaxial leaf epidermis of *N. nuda* plantlets cultivated in vitro under different light regimes. Abbreviations: W, white light; BR, blue–red light with high intensity; BRS, blue–red light with low intensity; st, stoma; pec, pavement epidermal cell; ngt, non-glandular trichome; gt I, II, III, glandular trichomes of Types I, II, and III, respectively. Scale bar: 50 µm.

The leaf epidermis of *N. nuda* plants cultivated in vitro under different LED light regimes showed irregularly shaped pavement cells, diacytic stomata and non-glandular and glandular trichomes (Figure 5).

The leaves were hypostomatic, with stomata predominantly located on the abaxial (lower) surface and only occasionally present on the adaxial (upper) surface. Stomatal frequency was strongly influenced by light treatment. Under BR light, stomatal density increased on both leaf surfaces compared to W and BRS light. The effect was most pronounced on the adaxial surface, where stomatal number under BR was approximately fivefold higher than under the other treatments (Table 2).

Trichome diversity and distribution were also examined. Non-glandular trichomes were uniseriate and multicellular (2–4 cells), typically with a swollen basal cell and a verrucose apical cell. Three types of glandular trichomes were observed: (1) Type I—long capitate trichomes (~74 μm in length), consisted of a 2–4-celled stalk, a swollen basal cell (~41 μm), a short subapical cell (~8 μm) and a pear-shaped head cell (~25 μm long, ~16 μm wide); (2) Type II—short capitate trichomes with a short stalk and a bicellular head (~25 μm long, 16 μm in wide); and (3) Type III—peltate trichomes with a short stalk and a four-celled glandular head (~50 μm in diameter) (Figure 5).

Type II glandular and non-glandular trichomes were observed on both epidermal surfaces, especially along veins and margins. Type III glandular trichomes were predominantly found within the areolae of the abaxial side, whereas Type I trichomes occurred sporadically on both surfaces.

Quantitative analysis showed that the frequencies of most trichome types varied between the upper and lower leaf surfaces. However, no significant differences were observed among the experimental treatments, except for Type III glandular trichomes. Their abundance under BR light was approximately double that recorded for W and BRS treatments (Table 3), suggesting that BR light promotes peltate trichome formation, potentially enhancing the plant’s defensive or secretory capacity.

#### 2.3.3. Photosynthetic Efficiency

**Figure 6:**
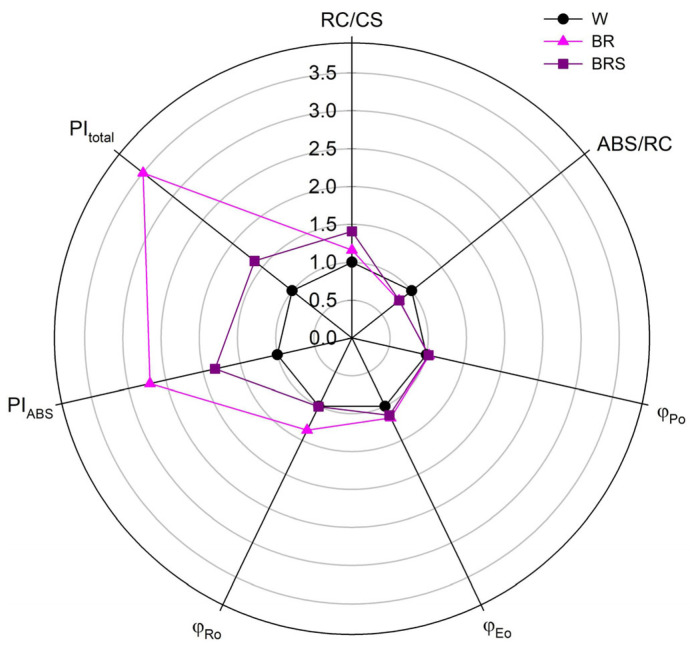
Relative chlorophyll a fluorescence parameters in leaves of *N. nuda* plants following ex vitro adaptation after in vitro growth under different light regimes. Values are presented relative to the W control. Abbreviations: W, white light; BR, blue–red light with high intensity; BRS, blue–red light with low intensity; RC/CS, active reaction centers per unit leaf area; ABS/RC, light flux absorbed per active reaction center; φ_Po_, maximum quantum yield of primary photochemistry in Photosystem II (PSII); φ_Eo_, quantum yield for electron transport beyond QA^−^ (from PSII to PSI); φ_Ro_, quantum yield for electron transport to the PSI end electron acceptors, relative to the total absorbed light; PI_ABS_, performance index based on absorption, reflecting the overall productivity of PSII; PI_total_, total performance index integrating the efficiencies of PSII and PSI.

To assess whether blue–red light preconditioning affects the ex vitro adaptation potential of *N. nuda*, in vitro-cultivated plants were first transferred to a white light phytotron chamber for one month, followed by two months in a greenhouse (Figure S3). The photosynthetic performance of the adapted plants was evaluated via the JIP test based on chlorophyll a fluorescence parameters (Figure 6).

Plants pre-treated with either BR or BRS light regime exhibited enhanced photosynthetic activity compared to those grown under W light. Specifically, both BR and BRS treatments led to an increased density of active reaction centers per unit leaf area (RC/CS) and a decreased light absorption per active reaction center (ABS/RC), indicating more efficient energy use under blue–red light conditions. Moreover, improvements were noted in several key functional parameters of the electron transport chain. The efficiency of intersystem electron flow (φ_Eo_) and electron transfer to PSI end acceptors (φ_Ro_) was higher in BR-treated plants, suggesting enhanced overall photosynthetic performance. The combined performance index of both photosystems (PI_total_) was also markedly higher in the BRS variant, indicating a potential advantage of moderate blue–red light intensity for long-term photochemical efficiency and acclimation.

## 3. Discussion

Light is a crucial environmental cue that regulates plant growth, morphology, metabolism and defense. Our study demonstrates that the spectral composition and intensity of light, particularly blue–red LED illumination, profoundly influence the physiology and adaptive potential of *N. nuda* plants cultivated in vitro. High-intensity BR light enhanced biomass accumulation, leaf expansion and mesophyll development, while promoting protein and starch accumulation, as well as increasing the level of phenolic compounds and antioxidant capacity. These physiological improvements were associated with specific hormonal shifts, notably in auxins, jasmonates and ABA, suggesting a coordinated regulation of growth and stress responses.

### 3.1. Oxidative Stress Caused by BR Light Triggers Antioxidant Response of N. nuda

The data suggests that a more favorable balance between light intensity and spectral quality supports chloroplast function and pigment biosynthesis under reduced blue–red light intensity. The decline in the content of plastid pigments observed under BR light indicates that excessive light can disrupt components of the photosynthetic system and lead to the generation of excited molecules that interact with molecular oxygen to form ROS. Subsequently, ROS interact with lipids and trigger peroxidation and the release of MDA, a well-established biomarker of oxidative stress [18]. Interestingly, although BR light induced oxidative stress, as evidenced by increased lipid peroxidation, it also activated compensatory antioxidant responses. This aligns with the concept of “eustress”, where controlled ROS production functions as a signal to activate protective pathways [19]. Enhanced accumulation of phenolic antioxidants and increased DPPH scavenging activity under BR light suggest that *N. nuda* uses a light-induced redox signaling mechanism to strengthen its defense capacity. Notably, BR light also induced dehydration, consistent with the moderate water stress conditions that are typical of the natural habitats of *Nepeta* species [14]. Studies on in vitro-cultivated *Nepeta* species have demonstrated that polyethylene glycol (PEG)-induced dehydration causes an oxidative stress response linked to increased accumulation of rosmarinic acid and decreased nepetalactone biosynthesis [14,20].

At the hormonal level, BR light specifically promoted the accumulation of auxins and jasmonates—phytohormones known to synergistically regulate flowering, root–shoot balance and defense responses [21,22,23]. The increased levels of ABA, a hormone often associated with abiotic stress tolerance, likely reflect moderate dehydration and may contribute to the improved ex vitro adaptation observed in BR-treated plants.

### 3.2. Phytochemical Adjustment to Blue–Red Light

Blue–red light treatment led to an overall increase in the total content of primary metabolites. Of particular interest is the modulation of volatile secondary metabolites in response to different light regimes. While BR light enhanced the biosynthesis of several monoterpenes and sesquiterpenes with potential ecological roles (1,8-cineole/eucalyptol, germacrene D, caryophyllene), it reduced the level of 4a-α,7-β,7a-α-nepetalactone, a compound known for its herbivore-deterring properties. Similar reductions in nepetalactone levels have been observed under drought stress [20], and the high-intensity BR illumination also triggered water loss. These findings suggest a potential trade-off between stress resilience and the production of specialized metabolites. Previous field studies on *N. nuda* volatiles demonstrated that elevated temperature enhanced the production of 1,8-cineole/eucalyptol, *trans*-caryophyllene and germacrene D, while precipitation and solar radiation had no significant effect [24]. On the other hand, the allelopathic activity of 1,8-cineole/eucalyptol [25,26] supports a scenario in which *N. nuda* may suppress the growth of neighboring plants (directly or indirectly by inhibiting growth-promoting soil bacteria) to secure access to nutritional resources. The increased accumulation of phenolic compounds under BR light further supports a coordinated response that includes antioxidant activity, allelopathy and photoprotection [15].

### 3.3. Blue–Red Light Modulates Leaf Structure and Enhances N. nuda Resilience to Stress

The increased stomata number and thickness of the palisade and spongy parenchyma under BR light aligns with previous reports that link light intensity to chloroplast density and CO_2_ assimilation efficiency in sun-adapted plants [27,28]. The elevated RC/CS and enhanced φ_Eo_ and φ_Ro_ values observed in BR- and BRS-pretreated plants further support the hypothesis that blue–red light primes the photosynthetic machinery for superior performance under ex vitro conditions.

Previous studies have shown that nepetalactone biosynthesis and accumulation in *N. racemosa*, *N. cataria* and *N. rtanjensis* occur in the peltate glandular trichomes of the leaves [13,20,29]. The increased formation of peltate trichomes in *N. nuda* under BR light may reflect a preparatory mechanism, either to compensate for reduced nepetalactone production, to secrete protective compounds under high light intensity, or alternatively, to provide localized shading.

Under the lower-intensity BRS light regime, plant height increased, and the leaf thickness decreased, indicating a potential shade-avoidance response. The fact that BRS treatment retained several BR-like effects, such as improved photosynthetic indices and volatile production, but with a reduced oxidative cost, suggests that low-intensity BR light may provide a more balanced strategy for the pre-acclimation of micropropagated plants. Alternatively, BR illumination could be applied for a shorter period, such as during the final two weeks of cultivation.

## 4. Materials and Methods

### 4.1. Plant Material

In vitro plants were maintained in the collection of the Department of Plant Physiology at the Faculty of Biology, Sofia University [15]. The plantlets were grown under sterile controlled conditions on standard Murashige and Skoog (MS) medium (MS, Duchefa, Haarlem, The Netherlands; ref. [30]) supplemented with 3% sucrose and 0.7% agar (Fluka Analytical, Munich, Germany). Cultivation was carried out under white light (80 μmol m^−2^ s^−1^ photosynthetically active radiation, PAR; warm white fluorescent, MASTER TL-D Super 80 36W/840 1SL/25, Philips, Pila, Poland) with a photoperiod of 16 h light/8 h dark, at 25 ± 1 °C and relative humidity of 60–70%. Plants were subcultured every 4 weeks using shoot tips.

### 4.2. Light Regimes and Treatment

**Figure 7:**
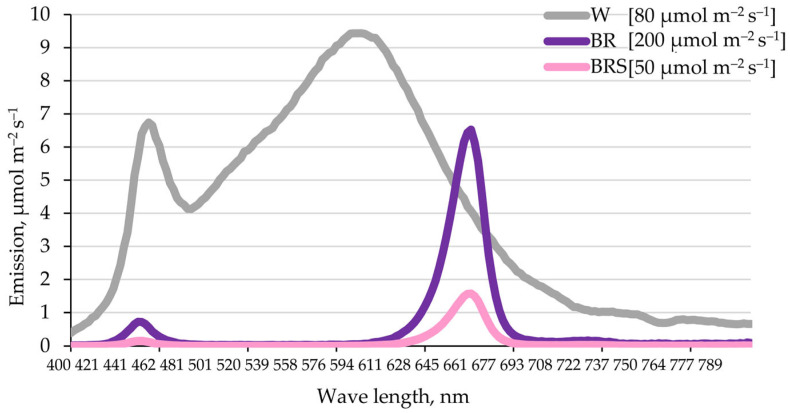
Emission spectra of the light sources used for in vitro cultivation of *N. nuda.* W—white light; BR—blue–red light with high intensity; BRS, “blue–red with shadow”—blue–red light with low intensity.

Three different light regimes were applied to evaluate the effects of spectral composition and intensity on *N. nuda* development. As a control, white (W) fluorescent light (80 μmol m^−2^ s^−1^ PAR) was used. Experimental treatments included two intensities of combined blue–red LED light (blue/red/far-red = 15%:75%:10%, OSRAM Opto Semiconductors, Regensburg, Germany): a high-intensity variant (BR, 200 m^−2^ s^−1^ PAR) and a low-intensity variant (BRS, 50 m^−2^ s^−1^ PAR), the latter also referred to as ‘shadowed’. The spectral profiles of the light treatments are presented in Figure 7.

Internode explants were cultivated in vitro under each light condition for 5 weeks under the conditions described above. After this period, newly-formed shoots were harvested and analyzed. Each treatment included a minimum of 20 biological replicates (*n* ≥ 20).

### 4.3. Morphometric Parameters

Dry biomass (dry weight, DW) was determined after drying plant samples in the dark at room temperature until a constant weight was achieved. Water content was calculated using fresh weight (FW) and DW and expressed as a percentage using the following formula:

$$Water\;content\;(\%)=\frac{\left(FW-DW\right)}{FW}\cdot 100$$

Shoot length of each plant was measured with a standard ruler. To estimate leaf area, fully developed leaves from the middle part of each plant were photographed alongside a scale. Leaf size was then calculated using ImageJ software (version 1.4.3, https://imagej.net/ij/, accessed on 21 July 2025)) [31].

### 4.4. Localization of ROS in Plants

The intracellular accumulation of reactive oxygen species (ROS) in *N. nuda* tissues was visualized using the fluorescent dye 2′,7′-dichlorofluorescein diacetate (DCF-DA), following the protocol by Yakimova et al. [32]. The fluorescent signal was observed under an LED epifluorescence microscope (excitation 460–470 nm; MRC Scientific Instruments, Harlow, UK) equipped with a DV-300 camera and LissView software (version 6.1.4.1).

The presence of specific ROS types—hydrogen peroxide (H_2_O_2_) and superoxide anion (O_2_^•−^)—was assessed using 3,3′-diamino benzidine (DAB) and nitroblue tetrazolium (NBT), respectively [33]. For NBT staining, the plant material was incubated for 6 h with a 0.05% NBT solution [0.025 g (or ½ tablet) NBT in 50 mL of 50 mM sodium phosphate buffer, pH 7.4]. For DAB staining, samples were incubated for 6 h in a solution of 1 mg mL^−1^ DAB in 10 mM Na_2_HPO_4_ containing 0.05% (*v*/*v*) Tween 20. All incubations were performed at room temperature in the dark with constant agitation (80 rpm). After staining, samples were cleared in a destaining solution (ethanol/acetic acid/glycerol = 3:1:1) and subsequently observed and photographed.

### 4.5. Simultaneous Extraction and Analysis of Metabolites

Analyses of pigments, free sugars, starch, malondialdehyde (MDA), total amino acids, phenols and flavonoids were performed according to the protocols described by López-Hidalgo et al. [34], collectively referred to as the “rainbow protocol”.

Fresh frozen plant material (*n* ≥ 10) was homogenized in liquid nitrogen, and 150 mg of the homogenate was resuspended in 1.5 mL of cold 80% ethanol, followed by ultrasonication for 10 min. The mixture was then centrifuged at 14000 rpm for 10 min, and the resulting supernatant (plant extract) and pellet were collected separately for subsequent analyses.

#### 4.5.1. Plastid Pigments

Pigment content in the ethanolic extract was determined spectrophotometrically by measuring absorbance at 470, 649 and 664 nm. Concentrations were calculated using the following equations:

$$Chlorophyll\;a\left(Chla\right)\left(\frac{\mu g}{mL}\right)=13.36\;A664-5.19\;A649$$

Chlorophyll b(Chlb)(μg/mL)=27.43 A649−8.12 A664

Carotenoids (μg/mL)=(1000A 470−2.13 Chla−97.63 Chlb)/209

Final pigment concentrations were expressed as milligrams per gram of FW (mg g^−1^ FW).

#### 4.5.2. Amino Acids

To quantify free amino acids, 300 μL of plant extract was mixed with 150 μL of 2% ninhydrin solution. The reaction mixture was incubated at 100 °C for 10 min in a thermoblock, then immediately cooled on ice. Subsequently, 750 μL of 96% ethanol was added to stabilize the color complex. Absorbance was measured at 440, 520 and 570 nm. Free amino acid content was calculated using a standard curve generated with L-proline and L-glycine, and the results were expressed as mg g^−1^ FW.

#### 4.5.3. Proteins

Total protein content and SDS-PAGE protein profiling were performed according to the protocol described by Chaneva et al. [35].

#### 4.5.4. Soluble Sugars

To 10 μL of plant extract, 600 µL of 0.1% Anthron reagent was added. The reaction mixture was kept on ice to prevent premature reaction, and then incubated at 100 °C in a thermoblock for 10 min. After cooling on ice, absorbance was measured spectrophotometrically at 625 nm. Soluble sugar content was calculated using a glucose standard curve and expressed as mg g^−1^ FW.

#### 4.5.5. Starch

The pellet obtained from the ethanol extraction was used for starch quantification. It was resuspended in 30% perchloric acid and incubated for 60 min at 60 °C. After cooling on ice, the samples were centrifuged for 10 min. For analysis, 10 μL of the resulting supernatant was mixed with 40 μL of 80% ethanol and 600 μL of Anthrone reagent. The reaction mixture was incubated at 100 °C for 10 min, then cooled on ice. Absorbance was measured at 625 nm. Starch content was calculated using a glucose standard curve and expressed as mg g^−1^ FW.

#### 4.5.6. MDA

To evaluate lipid peroxidation, malondialdehyde (MDA) content was measured via the thiobarbituric acid (TBA) assay. Samples were divided into two sets. For the first set (positive reaction), 50 μL of plant extract was mixed with 500 μL of 0.5% TBA in 20% trichloroacetic acid (TCA). For the second set (negative reaction), 50 μL of plant extract was mixed with 500 μL of 20% TCA, and the final volume was adjusted to 1 mL with 450 μL of 80% ethanol. All tubes were incubated at 95 °C for 30 min in a thermoblock and then cooled on ice. Absorbance was measured at 440, 532 and 600 nm. MDA content was calculated using the following equations:

$$A\;=\;[({A532}_{+TBA}-{A600}_{+TBA})\;-\;({A532}_{-TBA}-{A600}_{-TBA})$$

$$B=[({A440}_{+TBA}-{A600}_{+TBA})\;\times \;0.0571$$

$$\mathrm{MDA}\;(\mathrm{nmol}/\mathrm{mL})=\;\left[(A\;–\;B)/157.000\right]\;\times \;10^{6}$$

Final values were normalized to sample weight and expressed as nmol g^−1^ FW.

#### 4.5.7. Total Phenolics

Total phenolic content was determined using the Folin–Ciocalteu method, as described by Singleton et al. [36]. The reaction mixture consisted of 30 μL of plant extract, 500 μL of 10% Folin–Ciocalteu reagent and 470 μL of 7.5% Na_2_CO_3_. After incubation in the dark at room temperature for 30 min, absorbance was measured at 765 nm. Phenolic content was calculated using a gallic acid standard curve and expressed as mg g^−1^ FW.

#### 4.5.8. Antioxidant Activity

Antioxidant capacity was assessed using the DPPH (2,2′-diphenyl-1-picrylhydrazyl) radical scavenging assay, following the protocol of Brand-Williams et al. [37]. The reaction mixture consisted of 80 μL of extract and 1920 μL of 0.06 mM DPPH solution prepared in methanol. Samples were incubated for 30 min at room temperature in the dark, and absorbance was measured at 515 nm. Antioxidant activity was calculated using a Trolox standard curve and expressed as mM g^−1^ FW.

### 4.6. GC-MS Analysis of Volatile Compounds

The analysis of volatile compounds was performed according to the procedure described in detail by Zaharieva et al. [16].

### 4.7. Plant Hormone Profiling

Plant hormones were analyzed using solid phase extraction (SPE), followed by liquid chromatography–tandem mass spectrometry (LC-MS/MS), as described by Zaharieva et al. [16].

### 4.8. Light Anatomy and Epidermis

For anatomical analysis, small leaf segments (4–5 mm^2^) were excised from the central part of 20 leaves per light treatment and fixed in 3% (*m*/*v*) glutaraldehyde in 0.1 M sodium phosphate buffer (pH 7.4) for 12 h at 4 °C. Transverse handmade sections (*n* ≥ 15) were mounted in glycerol and examined using an Amplival 4 light microscope (Carl Zeiss, Jena, Germany). High-resolution microphotographs (2560 × 1960 pixels, *n* ≥ 15) were taken with an EcoBlue digital microscope (model EC.1657, Euromex, Arnhem, The Netherlands) equipped with a 5.0 MP USB-2 camera at 400× magnification. Morphometric measurements were performed using ImageJ (National Institutes of Health, Bethesda, MD, USA), and included lamina thickness (LL), mesophyll (M), palisade parenchyma (PP), spongy parenchyma (SP) and the adaxial (AdE) and abaxial (AbE) epidermal layers. Results were expressed in micrometers (µm). Epidermal features were analyzed using fragments from the central region of five leaves per treatment. Samples were preliminary rinsed with distilled water, bleached and rinsed again before mounting in glycerol. Stomatal and trichome densities (number mm^−2^) were estimated from 30 measurements completed with the Amplival 4 light microscope for each light treatment. Statistical analyses were performed using one-way analysis of variance (ANOVA), followed by Tukey-b post-hoc test for mean comparison with SPSS software (version 26.0, IBM Corp., IBM, Sofia, Bulgaria).

### 4.9. Ex Vitro Adaptation

After five weeks of in vitro cultivation under W, BR and BRS light regimes, plants were transferred to a phytotron chamber (POL-EKO APARATURA SP.J.A. Polok-Kowalska KK 350 STD, 1400 W, Wodzisław Śląski, Poland) for a one-month acclimatization period, followed by an additional two months of adaptation in a greenhouse.

### 4.10. Chlorophyll a Fluorescence

Chlorophyll a fluorescence was measured to assess photosynthetic efficiency via the JIP test, which characterizes the performance of the photosynthetic electron transport chain. The same fluorescence parameters described by Zhiponova et al. [3] were evaluated.

### 4.11. Statistics

Three biological replicates (each replicate included a representative sample of about 10–20 plants randomly selected from different culture vessels to ensure variability) and three technical replicates were analyzed. To assess statistical differences between variants, one-way ANOVA followed by Holm–Sidak post-hoc test was performed using Sigma Plot 11.0 software (Systat Software GmbH, Inc., Erkrath, Germany). Differences were considered statistically significant at *p* < 0.05 and are indicated by different letters.

## 5. Conclusions

Taken together, our results support the hypothesis that blue–red light, particularly at high intensity, acts as a powerful modulator of morphophysiological and metabolic traits in *N. nuda*. Preconditioning with BR light enhances traits associated with stress resistance, including antioxidant potential, secondary metabolite accumulation and structural readiness for stress, making it a promising tool for improving ex vitro performance in this and potentially other medicinal plant species. BRS light also shows potential for practical application; however, a slight increase in intensity would be beneficial to improve leaf anatomical structure.

We assume that after selecting an appropriate *N. nuda* genotype [38], additional factors could be employed to further increase plant productivity. Light is an ecologically and economically advantageous elicitor; therefore, future studies are strongly encouraged to explore its effects, particularly in combination with optimized temperature, irrigation and soil composition.

## Figures and Tables

**Table 1:** Leaf morphometric parameters of *N. nuda* cultivated in vitro under different light regimes. Mean values ± SD (standard deviation) shown for each treatment. Different letters indicate statistically significant differences (*p* < 0.05, one-way ANOVA, Holm–Sidak test). Abbreviations: W, white light; BR, blue–red light with high intensity; BRS, blue–red light with low intensity.

Tissue Thickness, µm	W	BR	BRS
Leaf lamina	97.8 ± 9.4 ^a^	120.8 ± 10.5 ^b^	82.2 ± 8.0 ^c^
Mesophyll	71.6 ± 11.3 ^a^	89.8 ± 11.9 ^b^	56.4 ± 6.8 ^c^
Palisade parenchyma	30.2 ± 5.5 ^a^	38.4 ± 4.1 ^b^	23.0 ± 4.0 ^c^
Spongy parenchyma	40.9 ± 5.3 ^a^	51.8 ± 8.1 ^b^	34.5 ± 6.2 ^c^
Adaxial epidermis	13.0 ± 4.2 ^a^	12.8 ± 2.7 ^a^	11.6 ± 3.0 ^a^
Abaxial epidermis	10.9 ± 2.1 ^a^	10.2 ± 2.3 ^a^	9.9 ± 1.8 ^a^

**Table 2:** Stomatal frequency on the adaxial and abaxial surfaces of leaves from *N. nuda* plantlets cultivated in vitro under different light regimes. Mean values ± SD are shown for each treatment. Different letters indicate statistically significant differences (*p* < 0.05; one-way ANOVA, Holm–Sidak test). Abbreviations: W, white light; BR, blue–red light with high intensity; BRS, blue–red light with low intensity.

Stomata Frequency, Number mm^−2^	W	BR	BRS
Adaxial epidermis	6.22 ± 9.56 ^b^	29.12 ± 20.33 ^a^	4.02 ± 5.56 ^b^
Abaxial epidermis	180.72 ± 31.64 ^b^	239.96 ± 33.60 ^a^	160.84 ± 30.56 ^b^

**Table 3:** Trichome frequency on the adaxial and abaxial leaf surfaces of in vitro-cultivated *N. nuda* plantlets under different light regimes. Mean values ± SD are shown for each treatment. Different letters indicate statistically significant differences (*p* < 0.05; one-way ANOVA, Holm–Sidak test). Abbreviations: W, white light; BR, blue–red light with high intensity; BRS, blue–red light with low intensity.

Trichome Frequency, Number mm^−2^	W	BR	BRS
Adaxial epidermis			
Non-glandular	5.62 ± 5.69 ^a^	5.82 ± 7.6 ^a^	5.62 ± 5.91 ^a^
Glandular Type II	9.84 ± 6.98 ^a^	14.66 ± 13.30 ^a^	9.84 ± 7.50 ^a^
Glandular Type I	rare	rare	rare
Abaxial epidermis			
Non-glandular	5.62 ± 4.73 ^a^	9.24 ± 7.71 ^a^	7.03 ± 6.54 ^a^
Glandular Type III	4.62 ± 4.66 ^b^	8.23 ± 5.59 ^a^	4.42 ± 4.17 ^b^
Glandular Type II	13.45 ± 7.37 ^a^	18.67 ± 10.05 ^a^	14.66 ± 7.86 ^a^
Glandular Type I	3.01 ± 4.40 ^a^	0.60 ± 1.84 ^a^	1.81 ± 3.59 ^a^

## Data Availability

Data are contained within the current article and its Supplementary Materials.

## Supplementary Materials

The following supporting information can be downloaded at https://www.mdpi.com/article/10.3390/plants14152285/s1: Figure S1: Comparison of the effect on protein accumulation of in vitro *N. nuda* cultured under different light conditions; Figure S2: ROS accumulation in leaves of in vitro *N. nuda* cultured under different light conditions; Figure S3: Ex vitro adaptation after in vitro cultivation of *N. nuda*; Table S1: Volatile compounds identified in *N. nuda* samples using GC/MS analysis.

## Abbreviations

The following abbreviations are used in this manuscript:

ABA	Abscisic acid
BR	Blue–red light with high intensity
BRS	Blue–red light with low intensity (“BR with shadow”)
DCF-DA	Dichlorodihydrofluorescein diacetate
DPPH	2,2-diphenyl-1-picrylhydrazyl radical
GC/MS	Gas chromatography/mass spectrometry
LED	Light-emitting diode
MDA	Malondialdehyde
PAR	Photosynthetically active radiation
ROS	Reactive oxygen species
W	White light
