# Supplementary material for: Blue–Red LED Light Modulates Morphophysiological and Metabolic Responses in the Medicinal Plant Nepeta nuda
Source: Plants (Basel). 2025 Jul 24;14(15):2285. doi: 10.3390/plants14152285 (PMC12348697; doi:10.3390/plants14152285)
Supplement: Supplementary file 1 [file plants-14-02285-s001.zip › plants-3731301-supplementary.pdf]

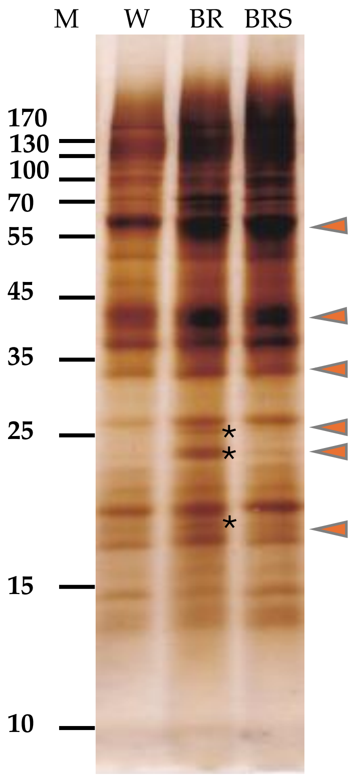

Figure S1. Comparison of the effect on protein accumulation of *in vitro N. nuda* cultured under different light conditions. SDS PAGE protein profiles are shown. Equal amount of extract was loaded onto each line. W – white light; BR – blue-red light with high intensity; BRS – blue-red light with low intensity. Arrowheads point to differences among the variants. Asterisk indicates specific protein abundance.

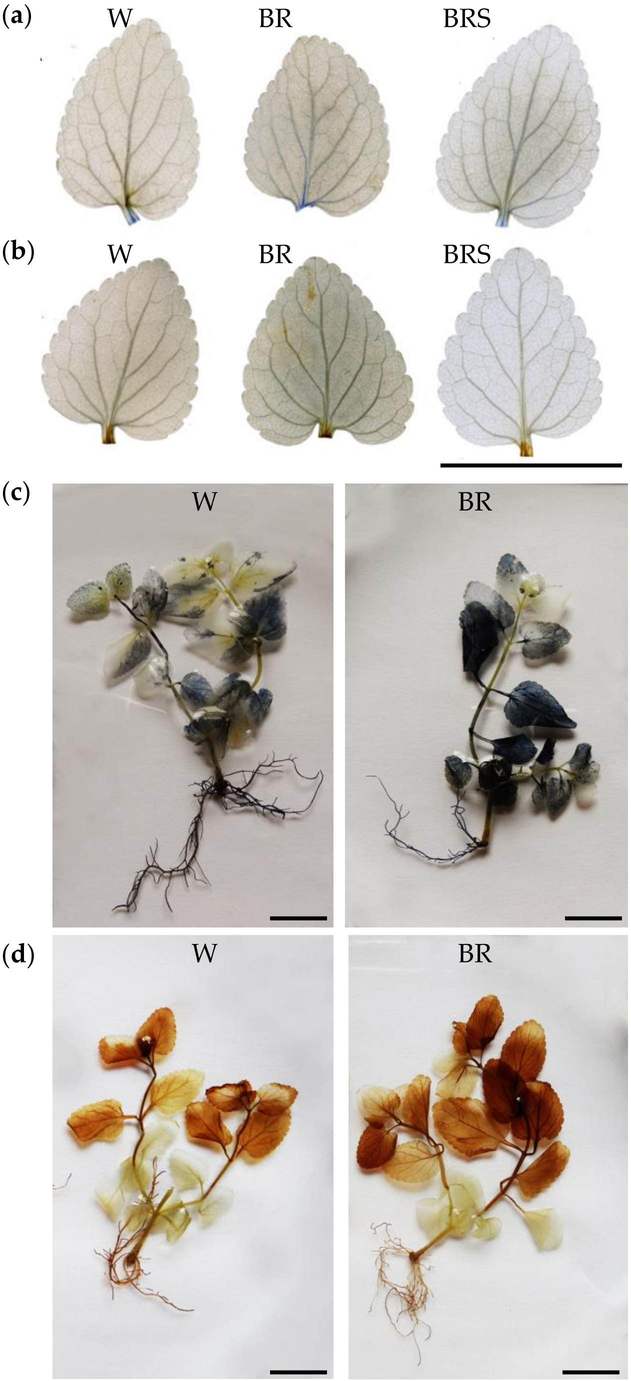

Figure S2. ROS accumulation in leaves of *in vitro N. nuda* cultured under different light conditions. In (a) and (b), the explants were grown for five weeks under W, BR and BRS lights. In (c) and (d), the first two weeks the plants were grown under normal W light, and then transferred to W and BR irradiation for two weeks. (a, c) Detection of superoxide anion (O2•−) determined by blue staining with nitroblue tetrazolium (NBT). (b, d) Detection of hydrogen peroxide (H_2O_2) determined by brown staining with 3,3′ diaminobenzidine (DAB). W – white light; BR – high intensity blue-red light; BRS – low intensity blue-red light. Scale bar: 1 cm

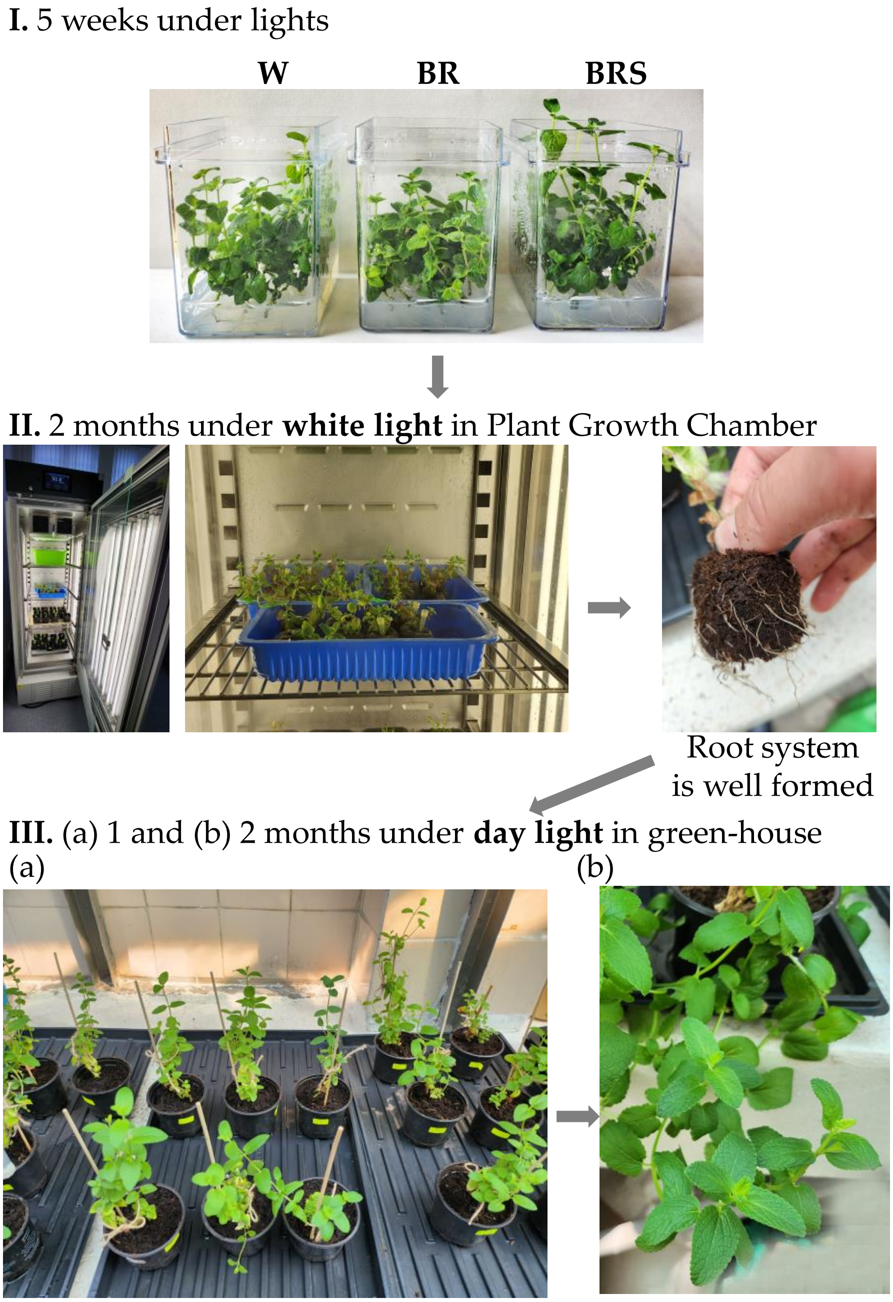

Figure S3. *Ex vitro* adaptation after *in vitro* cultivation of *N. nuda*.

Table S1. Volatile compounds identified in *N. nuda* samples using GC/MS analysis. Analysis includes shoots of *in vitro*-cultivated plants cultivated under different light variants. Heat map illustrates fluctuations among the plant variants for each compound (maximal in red). Statistical differences among variants were determined using one-way ANOVA (Holm–Sidak test), as different letters denote significant variations. The most abundant compounds are displayed in bold. RT-retention time; RI-retention index.

	RT	RI	**W**	**BR**	**BRS**
β-Pinene	10.18	1110.5	0.54[a]	**2.72**[b]	0.24[a]
Sabinene	10.53	1123.4	0.24[a]	**1.31**[b]	0.12[a]
Myrcene	11.75	1167.9	0.18[a]	**0.93**[b]	0.06[a]
D-Limonene	13.01	1209.9	0.06[a]	**0.41**[b]	0.03[a]
**1,8-Cineole/Eucalyptol**	**13.34**	**1218.3**	**4.20**[a]	**22.20**[b]	**1.88**[a]
trans-β-Ocimene	14.21	1242.2	0.06[a]	**0.26**[b]	0.06[a]
β-Ocimene	14.87	1259	0.34[a]	**1.39**[b]	0.31[a]
γ-Elemene	23.99	1489.8	0.05[a]	**0.43**[b]	0.00[a]
α-Gurjunene	25.99	1539	0.08[ab]	**0.59**[a]	0.00[b]
β-Copaene	27.74	1582.4	0.34[a]	**0.65**[b]	0.42[a]
β-Elemene	28.40	1598.7	0.51[a]	**1.05**[b]	0.61[a]
**Caryophyllene**	**28.71**	**1606.4**	**2.83**[a]	**4.74**[b]	**3.62**[ab]
Humulene	31.52	1680.8	0.61[a]	**0.91**[b]	0.74[ab]
δ-Terpineol	31.67	1684.9	0.10[a]	**0.61**[b]	0.05[a]
α-Terpineol	32.61	1709.7	0.37[a]	**1.90**[b]	0.19[a]
**Germacrene D**	**33.02**	**1720.6**	**6.66**[a]	**13.38**[b]	**8.55**[a]
Bicyclogermacren	33.95	1745.2	0.37[a]	**1.87**[b]	0.21[a]
Dimethyl sulfone	40.53	1929.4	0.21[a]	0.16[a]	0.27[a]
**4a-α,7-β,7a-α-Nepetalactone**	**45.50**	**2078.8**	**63.57**[ab]	**31.89**[a]	**70.00**[b]
